# Metacaspases: Potential Drug Target Against Protozoan Parasites

**DOI:** 10.3389/fphar.2019.00790

**Published:** 2019-07-18

**Authors:** Vandana, Rajnikant Dixit, Rajnarayan Tiwari, Anju Katyal, Kailash C. Pandey

**Affiliations:** ^1^Host-Parasite Interaction Biology Group, ICMR-National Institute of Malaria Research, New Delhi, India; ^2^Dr Ambedkar Center for Biomedical Research, Delhi University, New Delhi, India; ^3^Department of Biochemistry, ICMR-National Institute for Research in Environmental Health, Bhopal, India

**Keywords:** malaria, metacaspases, druggable target, proteases, plasmodium

## Abstract

Among the numerous strategies/targets for controlling infectious diseases, parasites-derived proteases receive prime attention due to their essential contribution to parasite growth and development. Parasites produce a broad array of proteases, which are required for parasite entry/invasion, modification/degradation of host proteins for their nourishment, and activation of inflammation that ensures their survival to maintain infection. Presently, extensive research is focused on unique proteases termed as “metacaspases” (MCAs) in relation to their versatile functions in plants and non-metazoans. Such unique MCAs proteases could be considered as a potential drug target against parasites due to their absence in the human host. MCAs are cysteine proteases, having Cys-His catalytic dyad present in fungi, protozoa, and plants. Studies so far indicated that MCAs are broadly associated with apoptosis-like cell death, growth, and stress regulation in different protozoa. The present review comprises the important research outcomes from our group and published literature, showing the variable properties and function of MCAs for therapeutic purpose against infectious diseases.

## Introduction

**Figure 1:**
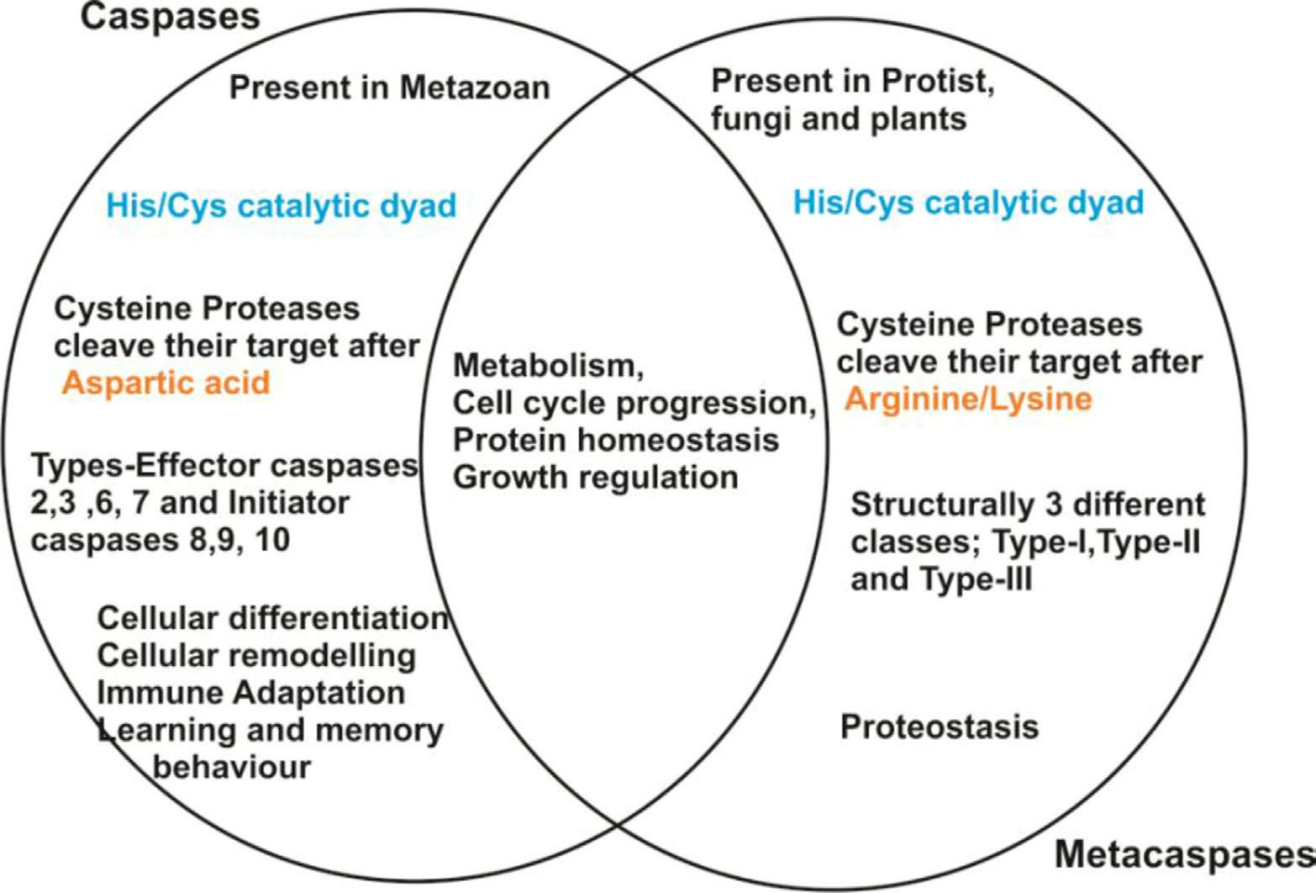
Summarized pictorial representation showing the key properties of caspases and metacaspases.

Studies over the past 15 years demonstrated that the proteases are not only essential for the maintenance of normal physiology but also play important roles in the regulation of cellular homeostasis and metabolism. Several proteases are considered as a potential drug target against the majority of infectious diseases. For an instance, antiretroviral drugs that inhibit viral integrase or GP41 (trans-membrane protein) have been clinically approved to treat HIV infection (Arhel and Kirchhoff, 2010). Additionally, a broad network of protein–protein interactions was elucidated for treating various types of cancer (Kanhaiya et al., 2017) and other diseases like osteoporosis (Wnt proteins) (Rochefort, 2014). Similarly, several malaria parasite proteases have been largely targeted for their role in pathogenesis. Recently, plasmepsins (PMs), particularly PM-V, IX and X of *Plasmodium falciparum*, receive prime attention for exploring their potential as drug targets (Russo et al., 2010; Nasamu et al., 2017). Further, unique proteases called “metacaspases” are also presently focused for their role in programmed cell death (PCD) of the protozoan (Uren et al., 2000). However, MCAs are structurally related to metazoan caspases, having Cys-His catalytic dyad but possess different substrate specificity (Uren et al., 2000; Tsiatsiani et al., 2011). Metacaspases have a highly acidic S1 pocket leading to arginine and lysine specificity at the P1 position, in lieu of aspartic acid specificity for caspases (Mottram et al., 2003; Atkinson et al., 2009) (**Figure 1**). Further, paracaspases are also caspase-related proteins found in metazoans and *Dictyostelium* (Uren et al., 2000; Atkinson et al., 2009).

It has been proposed that metacaspases and paracaspases should be separated in a specific family of clan CD. The basic topology of the caspases (C14A) and paracaspases (C14B) is found to be similar, whereas the topology of metacaspases (C14B) is quite different, suggesting that metacaspases were not similar with the caspases and paracaspases (Vercammen et al., 2004; Hachmann et al., 2012; Hulpiau et al., 2016). As per the present MEROPS classification, (Rawlings et al., 2017), all known cysteine proteases are categorized into 14 diverse clans according to their tertiary structure. Based on sequence similarity, proteases are clustered into families within each clan. Therefore, caspases, metacaspases, and paracaspases fit into the same clan of proteases (CD clan) and also to the same family (C14) based on the sequence similarity. The clan CD consists of cysteine-dependent proteases with a unique α/β-fold called caspase-hemoglobinase (CHF) fold, which contains a large (p20) subunit having the catalytic histidine/cysteine dyad and a small (p10) subunit (Walker et al., 1994). There are three motifs in most of the conserved parts of the CHF fold: one at the N-terminal β-strand, one before the catalytic histidine, and the other motif before the catalytic cysteine (Aravind and Koonin, 2002). The evolution of proteases from C14 family covered a long and complex series of speciation and duplication events that resulted in the significant variation in biochemical properties and hence a high degree of functional divergence. This notion was particularly true for metacaspases, which exhibited broader structural variation compared to caspases and paracaspases (Minina et al., 2017).

The comparative analysis of the different properties of caspases, metacaspases, and paracaspases reveals similarities and differences among them (**Table 1**).

**Table 1:** Comparative analysis of key properties of caspases, metacaspases, and paracaspases.

Properties	Caspases	Metacaspases	Paracaspases	Reference
*Distribution*	Metazoans	Protists, fungi, algae, and plants	Metazoans and *Dictyostelium*	Uren et al. (2000)
*Catalytic site*	His-Cys catalytic dyad	His-Cys catalytic dyad with few exceptions such as *Tb*MCA-1 and *Tb*MCA-4 that have Tyr and Ser in place of His-Cys.	His-Cys catalytic dyad	Uren et al. (2000); Hachmann et al. (2012); McLuskey and Mottram (2015)
*Substrate specificity*	Aspartic acid specificity	Arginine and lysine substrate specificity	Arginine-specific protease	Uren et al. (2000); Hachmann et al. (2012)
*Types/forms*	Effectors caspases (caspase-2, -3, -6, -7) and initiator caspases (caspase-8, -9, -10)	Type-I metacaspases have N-terminal pro-domain with proline-rich repeat motif and zinc finger motif. Type II metacaspases lack pro-domain but possess a linker region between the large (p20) and small (p10) subunits. Type III metacaspases found only in algae that have undergone secondary endosymbiosis	Type-1 paracaspases constitute MALT1-like domain having death domain, immunoglobulin-like domains and a caspase-like domain. Type-II paracaspases in metazoan represent ancestral form, having caspase-like-domain.	Uren et al. (2000); Mottram et al. (2003); Elmore (2007); Hulpiau et al. (2016)
*Biological functions*	Key regulators of programmed cell death, proliferation and inflammation, playing essential roles in the survival and death of animal cells.	Multifunctional proteases essential for parasite physiology but their detailed functions were poorly characterized.	Plays a major role in several pro-inflammatory pathways in innate and adaptive immunity.	Nuñez et al. (1998) Elmore (2007)
*Enzymatic functions*	Endo-proteases- hydrolyze peptide bonds that depend on catalytic cysteine residue in the active site and occur after aspartic acid residue in the substrates. Caspase-mediated processing results in substrate inactivation. It may also generate active signaling molecules that participate in ordered processes such as apoptosis and inflammation.	Cysteine proteases hydrolyze peptide bonds after arginine/lysine residues in their substrates Report on *T. brucei* MCA-4 suggested that the phenotypes induced by *Tb*MCA-4 expression in yeast were completely lost when the putative catalytic dyad residues histidine_164_ and cystein_218_ were both independently mutated to alanine.	Cysteine proteases hydrolyze peptide bonds after arginine residues in their substrates	1. Uren et al. (2000); Szallies et al. (2002); McIlwain et al. (2013)

## Evolutionary Diversity of Metacaspases-Like Proteases

Evolutionary distribution of metacaspases among different phyla indicated that such proteases were evolved through endosymbiotic gene transfer (EGT) (Choi and Berges, 2013). Based on structural differences, metacaspases were subdivided into types I, II, and III. Type I metacaspases are defined by presence of an N-terminal extension and a zinc-finger motif (Vercammen et al., 2004), while type II category contains a linker domain, which separates the p20 and p10 domains. Moreover, type III metacaspases have unusual rearrangement of the two domains, with p10 domain located N-terminally to the catalytic p20 domain (Klemenčič and Funk, 2019). Although the present classification of metacaspases was based on the presence of structural features (e.g., an N-terminal domain for type I and a discrete linker region for type II metacaspases), various subtypes of metacaspases have arisen during evolution. For example, N-terminal domain of type I metacaspases is absent in prokaryotes and secondary endosymbionts, while the calcium-independent type II metacaspases comprise a shorter linker region between the p20 and p10 domains (Klemenčič and Funk, 2019). Type I and II metacaspases have been identified in plants based on their domain structures and similarities with metazoan “initiator” and “executioner” caspases. MCAs of type III were present only in algae that underwent secondary endosymbiotic processes; additional metacaspase-like proteases are reported in the Glaucophyta and Rhodophyta (Choi and Berges, 2013) and photosynthetic bacteria (Asplund-Samuelsson et al., 2012), indicating that they are the evolutionary ancestor of the advanced metacaspases. Further, metacaspase-like proteases in phytoplankton showed sequence homology with other metacaspases, but defies classification in conventional schemes; rather such metacaspase-like proteases exist in bacteria alongside a variant of type I metacaspases (Atkinson et al., 2009). Type II and III metacaspases were not detected in bacteria and they might be variants of bacterial type I metacaspases that evolved in plants and phytoplanktonic protists, respectively, during the establishment of plastids through the primary and secondary endosymbiotic events (Choi and Berges, 2013). From an evolutionary perspective, the existence of multiple metacaspases with structural and functional differences could be of great interest as it could be used to trace the origin of the multiple roles fulfilled by the closely related caspases in metazoans (**Table 2**).

**Table 2:** Structural–functional analyses of caspases, metacaspases, and paracaspases.

Caspases	Metacaspases	Paracaspases
• Cysteine-dependent, aspartate-specific peptidase, • The effector caspases have short pro-domains (approx. 25 residues), whereas inflammatory and initiator caspases have long pro-domains (approx. 100–200 residues), which contain either CARD (caspase recruitment domain–inflammatory and initiator caspases) or DED (death effector domain-initiator caspases) motifs (Klemenčič and Funk, 2018) • Inflammatory/initiator caspases recognized the cell death stimuli and get activated, which in turn leads to activation of effector caspases, which ultimately causes the cell death.	• Cysteine-dependent, arg/lys-specific peptidase • X-ray crystal structure of an inactive mutant of TbMCA2 (TbMCA2 C213A) revealed a core caspase fold with an eight-stranded β-sheet that stabilized the enzyme as a monomer and a well-ordered N-terminus, which wrapped around the molecule covering the catalytic dyad. It is structurally homologous to β5 in the caspases—denoted as ^H^β5 (^H^5) (McLuskey et al., 2012) • Structural–functional correlation of these proteases not known in detail. However, these are multifunctional in nature, responsible for regulation of stress, metabolism and cell death of the parasite.	• Caspase subfamily twC14B, arginine specific peptidase • Para-caspase structures come from the human and murine mucosa-associated lymphoid tissue translocation protein 1 (MALT1). • The full-length protein comprises an N-terminal death domain (DD), followed by two immune-globin (Ig)-like domains (Ig1 and Ig2), the paracaspase domain, a further Ig-like domain (Ig3), and approximately 100 C-terminal residues with no apparent secondary structure (McLuskey and Mottram, 2015) • Structurally, 79% similar to caspase-7. • Play the central role in the activation of lymphocytes and other immune cells (Jaworski and Thome, 2016).

## Distribution and Functional Specification of Parasite Metacaspases

The current ongoing research on metacaspases has brought information about their biological functions in protozoa and plants (Vercammen et al., 2004; Meslin et al., 2011; Rathore et al., 2015; Peña et al., 2017). Unequal distribution of metacaspases between different phyla is an important paradigm to describe their multi-functionality such as their role in cell death, stress regulation, growth and development of parasite, etc.

### *Trypanosoma* Metacaspases

#### Trypanosoma Brucei

**Figure 2:**
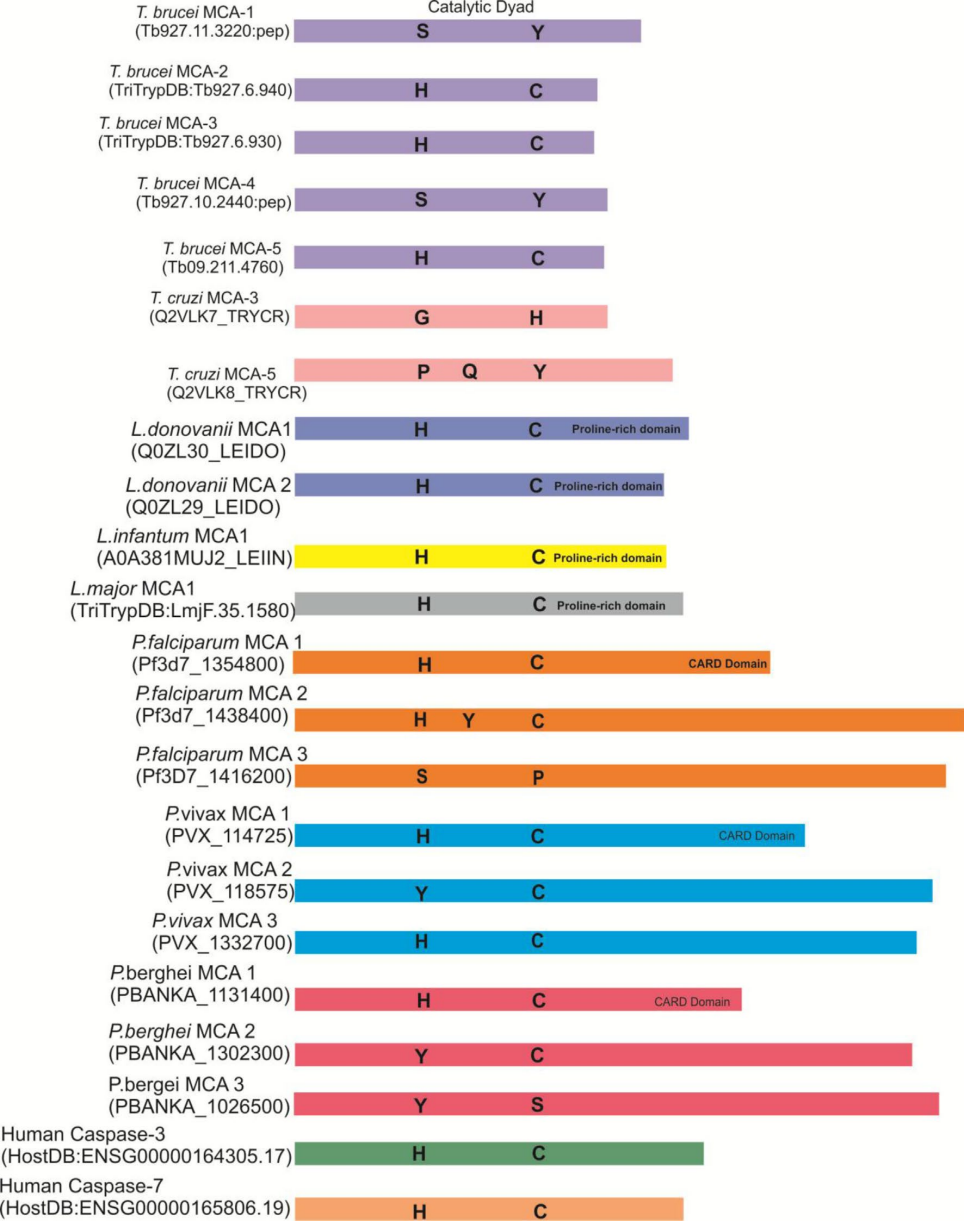
Representation of the relative sizes and the predicted catalytic residues of metacaspases from protozoan and human caspases 3 and 7. In *Tb*MCA-1 and 4, catalytic dyad His/Cys were subjected to be replaced by Ser/Tyr, respectively. Similarly, in *Pf*MCA-3, His/Cys catalytic dyad is replaced by ser/pro residues.

**Figure 3:**
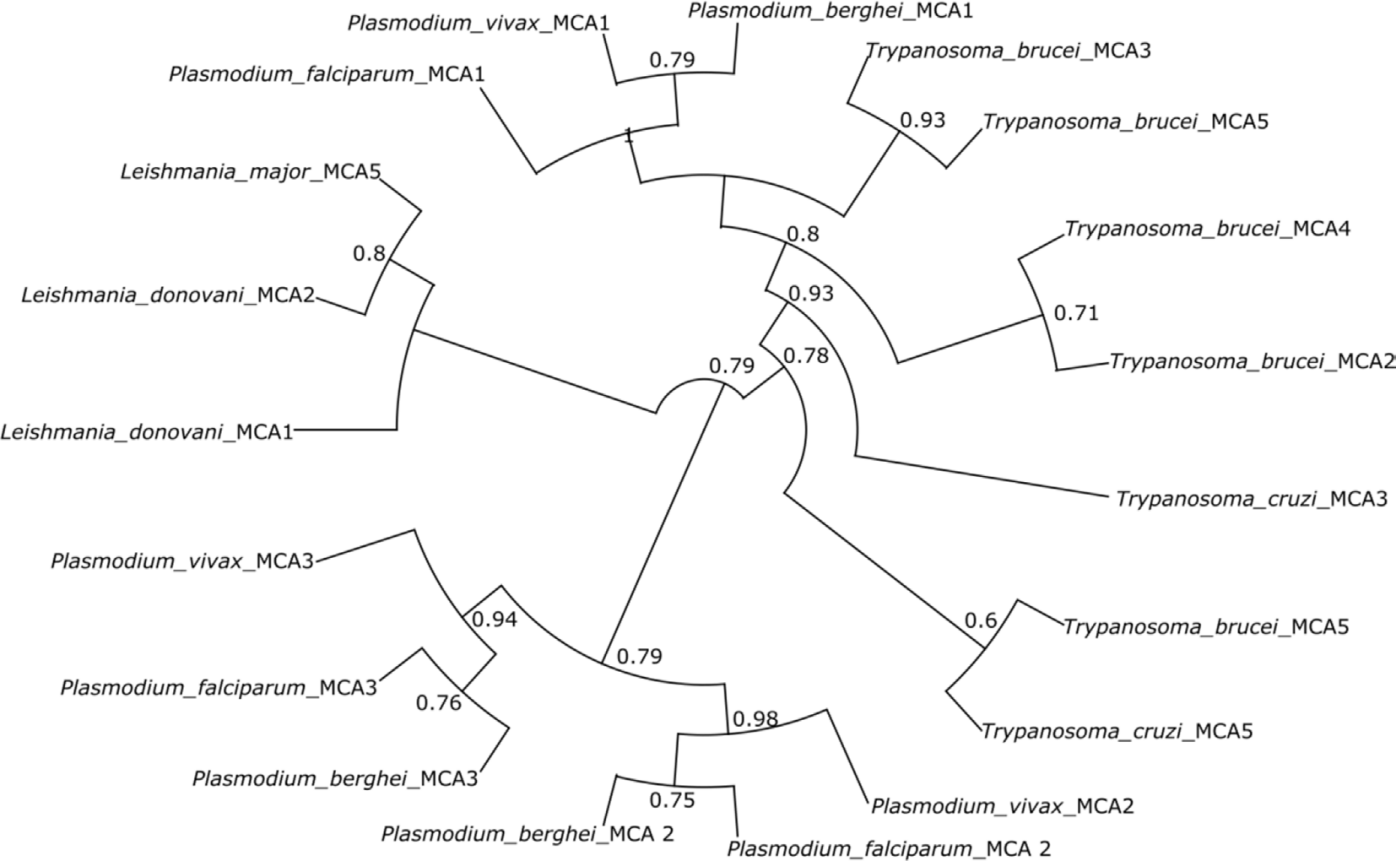
Phylogenetic tree of metacaspases from protozoan parasites. The tree shows the distinct relationship between different metacaspases of the different parasites. The tree was constructed using the maximum parsimony method. *Plasmodium* metacaspases *Pf*MCA-2, 3, *Pb*MCA-2, 3, and *Pv*MCA-2, 3 are phylogenetically distinct from metacaspases-1 of *Plasmodium* and other parasite metacaspases.

**Figure 4:**
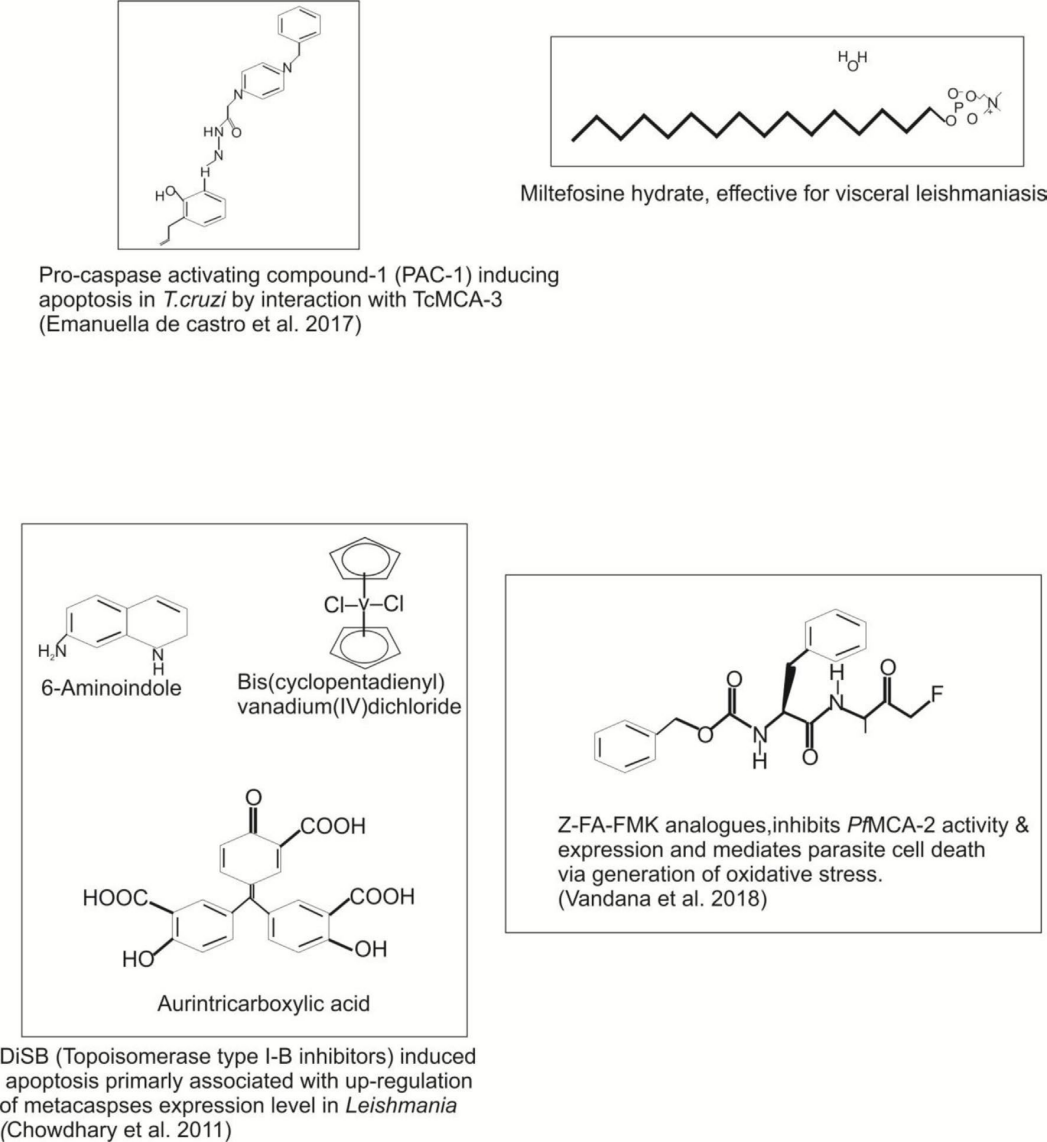
Potential inhibitors/compounds used to target the different protozoan metacaspases, which could be further exploited as an effective drug target against infectious diseases.

**Figure 5:**
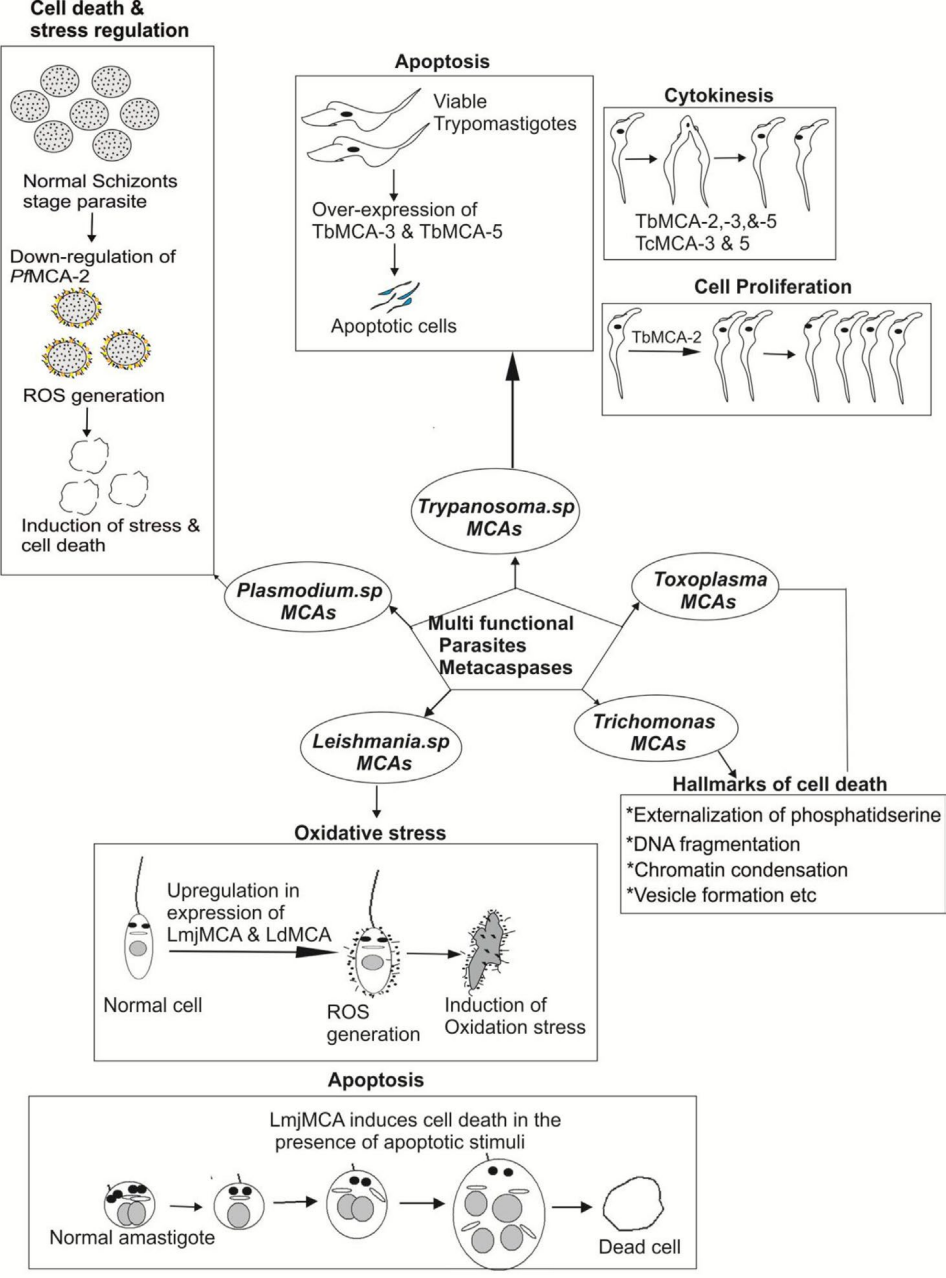
Pictorial depiction for multi-functions of metacaspases in different parasites: *Trypanosoma* metacaspases are found to play versatile function such as apoptosis, cell proliferation, cytokinesis of the parasites (Meslin et al., 2011; Proto et al., 2011); *Leishmania* metacaspases are involved in stress regulation and autophagy of the parasite (Khademvatan et al., 2011; Casanova et al., 2015; Peña et al., 2017); *Plasmodium* metacaspases also play a role in apoptosis and stress regulation of the parasites (Vandana et al., 2018).

The genome of *Trypanosoma brucei* encodes five metacaspases: *Tb*MCA1 to *Tb*MCA5 were similar with type- metacaspases and did not exhibit any processing under normal growth condition of parasite (Kosec et al., 2006) (**Figures 2**–**4**). The three corresponding metacaspases genes (*Tb*MCA-1, *Tb*MCA-2, and *Tb*MCA-4) of *T. brucei* seem to be absent in *Trypanosoma cruzi*. Studies suggested that *Tb*MCA-2, *Tb*MCA-3, and *Tb*MCA-4 were maximally expressed in the bloodstream form of the parasite than in the procyclic form except *Tb*MCA-5, which is expressed uniformly in both the forms of parasite life cycle (Meslin et al., 2011; Proto et al., 2011). A significant proportion of *Tb*MCA-2, *Tb*MCA-3, and *Tb*MCA-5 were located predominantly in endosomes associated with RAB11 (Ras-like GTPase responsible for intracellular transport and cytokinesis) (Engstler et al., 2004; Helms et al., 2006;McLuskey and Mottram, 2015). There are three *T. brucei* metacaspases, *Tb*MCA-2, *Tb*MCA-3, and *Tb*MCA-5, which are known to be active cysteine peptidases containing canonical histidine/cysteine dyad (Kosec et al., 2006; Moss et al., 2007), whereas *Tb*MCA-1 and *Tb*MCA-4 contain amino acid substitutions in their catalytic residues (histidine to tyrosine and cysteine to serine, respectively). On the other hand, a similar substitution of amino acid in *Tb*MCA-4 resulted in a catalytically inactive pseudopeptidase, but it was still functional as a membrane-linked virulence factor, which was processed by *Tb*MCA-3 (Meslin et al., 2011; Proto et al., 2011). The remaining genes (*Tb*MCA-1 and *Tb*MCA-4) were found to encode inactive proteases because they were predicted to have serine and tyrosine residues in place of catalytic cysteine and histidine residues, respectively (Meslin et al., 2011; Proto et al., 2011). Moreover, *Tb*MCA-5 has unusual proline, glutamine, and tyrosine-rich C-terminal extension in addition to its catalytic domain (Proto et al., 2011). On the other hand, *Tb*MCA-2 and *Tb*MCA-3share 89% sequence similarity, differing only at the N-terminus region. Studies on substrate specificity showed that *Tb*MCA-2 has arginine/lysine specificity at the P1 position and enzyme activity was strictly Ca^2+^-dependent, requiring 1 mM CaCl_2_ for maximum activity (Moss et al., 2007; Machado et al., 2013). Recent literature regarding the functionality of *T. brucei* metacaspases suggested that these proteases are likely to be involved in the regulation of PCD. For instance, Szallies et al. (2002) reported that the overexpression of *Tb*MCA-4 in yeast cells causes phenotypic changes in terms of growth retardation, loss of clonogenicity followed by loss of respiration competence in the yeast cells *in vitro* (Szallies et al., 2002). However, the phenotypes induced by *Tb*MCA-4 expression in the yeast cells were completely lost when the putative catalytic dyad residues histidine_164_ and cystein_218_ were both independently mutated to alanine. Yeast cells expressing the respective alleles of *Tb*MCA-4 were able to grow normally as the control (Szallies et al., 2002; Laverrière et al., 2012; McLuskey et al., 2014). This result clearly demonstrated that the effect of TbMCA-4 depends on the putative catalytic dyad residues (Szallies et al., 2002; McLuskey et al., 2014). Further, studies speculated that *Tb*MCA-2, *Tb*MCA-3, and *Tb*MCA-5 were involved in the regulation of cleavage furrow formation during cytokinesis of blood stream form (BSF) (Helms et al., 2006). However, the process of cytokinesis in BSF was not well illustrated, but it seems to be different from mammalian cells (Szallies et al., 2002; Machado et al., 2013). In addition, RNAi down-regulation study demonstrated that *Tb*MCA-4 was essential for cell proliferation of the parasite (Meslin et al., 2011) (**Figure 5**). Further, *Tb*MCA-4 processing by *Tb*MCA-3 established an important link between two MCAs and that could be an indication of the existence of MCAs cascade similar to the mammalian caspases cascade system (McLuskey et al., 2014). The structural studies of *Tb*MCA-2 suggested that metacaspases and caspases might be evolved independently from an ancestral metacaspase-like peptidase, with each family of enzymes evolving distinct activation mechanisms to regulate cell death pathways.

Conclusively, studies on *T. brucei* metacaspases suggested that its genome encodes MCA1-5 of which MCA-1 and MCA-4 lack the predicted active site cysteine and probably are not active cysteine peptidase. However, it cannot yet be ruled out that MCA-1 or 4 might compensate for the lack of MCA-2, -3, and -5 and carried out their role in mutant parasites. Indeed, it will be interesting to find out whether MCA-1 or -4 are up-regulated in MCA-2, -3, and -5 knockout lines. Moreover, it is tempting to hypothesize that MCA-2, -3, and -5 are involved in regulation of cleavage furrow formation during BSF cytokinesis, but little is known about the process of cytokinesis in BSF trypanosomes. Additionally, how the *Trypanosome* MCAs are directed to the compartment in which they reside is unclear. As per the literature, MCA-3 and MCA-5 are predicted to have signal peptides, whereas MCA-2 does not. Therefore, MCA-3 and MCA-5 are likely to be sorted through the classic secretory pathway, while MCA-2 might either be trafficked in association with MCA-5, MCA-3, or any other ER directed protein, or through an alternative secretory pathway. Further, analysis of *T. brucei* MCAs is crucial to illuminate their function and to provide insights into the regulatory networks to which both active and inactive MCAs participate. Whether they might be responsible for PCD-like phenomena is an open question. Although it has been anticipated that metacaspases of yeast (González et al., 2007) and plants (Sundström et al., 2009) have caspase-like functions linked with PCD, there have not been any reported evidence for similar functions in BSF *T. brucei*. It cannot yet be ruled out that metacaspases are involved in apoptotic-like cell death of the *T. brucei* BSF; however, the data available suggest that metacaspases have PCD-independent functions that might be associated with RAB11-positive endosomes. However, metacaspases also occur in vesicles lacking RAB11; the significance and more research on this await elucidation.

#### Trypanosoma cruzi

In *Trypanosoma cruzi*, two metacaspase genes were reported, namely, *Tc*MCA-3 and *Tc*MCA-5. The metacaspase genes of *T. cruzi* were found to be homologous with *Tb*MCA-3 and *Tb*MCA-5 of *T. brucei* (Proto et al., 2011). Moreover, approximately 16 copies of *Tc*MCA-3 and a single copy of *Tc*MCA-5 per haploid genome have been reported. Further, the His/Cys catalytic dyad was present in both *T. cruzi* metacaspases, but *Tc*MCA-3 showed a substitution of glycine→cysteine at the position next to the catalytic histidine residue. This glycine residue was broadly conserved among peptidases belonging to the clan CD; therefore, *Tc*MCA-3 mutants with glycine instead of cysteine might be an inactive peptidase, and predicted to have an only regulatory function (Hulpiau et al., 2016). The association of *Tc*MCAs with the RAB11 associated endosomes was not yet reported. Extending the role of *T. cruzi* metacaspases, there was indirect evidence suggesting that the metacaspases might be involved in PCD of the parasite (Helms et al., 2006). For an instant, epimastigotes over-expressing *Tc*MCA-5 were more sensitive to fresh human serum (FHS)-induced PCD than the controls (Proto et al., 2011). In addition, *Tc*MCAs also re-localized from the cytoplasm to the nucleus during apoptosis induced by FHS. Moreover, PCD was paralleled by an increase in peptidase activity against Z-YVAD-AFC (typical caspase substrate). The overexpression of *Tc*MCA-3 in human cell line did not induce death or morphological changes typical of apoptosis (Alvarez et al., 2012). On the other hand, overexpression of *Tc*MCA-5 in epimastigotes rendered them more susceptible to PCD, whereas *Tc*MCA-3 overexpression is found to be lethal to parasite (Hulpiau et al., 2016). Studies reveal that the antagonistic activities of *T. cruzi* metacaspases affect the balance between cell proliferation, death, and differentiation of the parasite (Laverrière et al., 2012). Recently, de Castro et al. (2017) demonstrated that the procaspase-activating compound 1 (PAC-1) induces apoptosis in *T. cruzi* by interaction with *Tc*MCA-3. The authors also revealed that the PAC-1 induces loss of cell viability, loss of mitochondrial potential, and externalization of phosphatidylserine (de Castro et al., 2017) (**Table 3** and **Figure 5**). Conclusively, the direct involvement of TcMCA-3 and -5 in cell death is an open debate as it was evident that PCD in response to exposure of FHS occurred in *T. cruzi*. This could be beneficial in many ways, for example, by preventing an early inflammatory response if epimastigote enters the human bloodstream or could be rendered host macrophages more susceptible to invasion. Nevertheless, whether cell death processes in *Trypanosoma* parasite are mediated by MCA proteases and similar biochemical mechanisms as in metazoan still remains an open subject.

**Table 3:** Comparative analyses of key features of protozoa metacaspases.

Features	*Leishmania* metacaspases	*Trypanosoma* metacaspases	*Plasmodium* metacaspases
*Cys/His Catalytic dyad*	All metacaspases contain His/Cys dyad.	*Tb*MCA2, *Tb*MCA3, *Tb*MCA5, *Tc*MCA5 and *Tc*MCA3 possess His/Cys dyad (McLuskey et al., 2014)	MCA-1 and -2 of *P. vivax, P. falciparum* and *P. berghi* have His/Cys dyad. *Pv*MCA-3 has Cys/His catalytic dyad, but it is absent in *Pf*MCA-3 and *Pb*MCA-3.
*Localization*/ *Expression*	*Ld*MC1 and *Ld*MC2 expressed in promastigote and axenic amastigote forms. *Lmj*MCA located in the mitochondrion and associated with the mitotic spindle (Castanys-Muñoz et al., 2012).	*Tb*MCA-2, *Tb*MCA-3, and *Tb*MCA-4 expressed in bloodstream stage only. *Tb*MCA-5 expressed in both life cycle stages. *Tb*MCA-2, *Tb*MCA-3, and *Tb*MCA-5 were located predominantly in endosomes associated with RAB11.	*Pf*MCA-1 and *Pv*MCA-1 gene expressed during asexual stages (Meslin et al., unpublished observations). *Pb*MCA-1 expression was detected in female gametocytes, oocysts, and sporozoites. *Pf*MCA-2 was localized in schizonts and gametocytes stages I–IV (Vandana et al., 2018).
*Enzymatic Activation/Processing*	*Ld*MCAs has arginine/lysine specificity without any proteolytic activation *Lmj*MCA activated by auto-processing and shows arginine/lysine substrate specificity.	Arg/Lys specificity and activity was strictly Ca^2+^-dependent.	Arg/Lys specificity and activity were Ca^2+^-independent and no auto-processing occurred in *Pf*MCA-2 (Vandana et al., 2018). *Pf*MCA-1 shows auto processing leading to pro-domain removal as typical of initiator caspases.
*Forms/Types*	All *Leishmania* species expressed one single metacaspase gene except *L.infantum* and *L*. *donovani*, in which two metacaspases were reported.	Type-I metacaspases; *T. brucei* has *Tb*MCA1-*Tb*MCA5 and *T. cruzi* has *Tc*MCA3 and *Tc*MCA5.	Type-I metacaspases; *P. falciparumPf*MCA1-3, *P. vivax Pv*MCA1-3, and P. berghei (*Pb*MCA1-3)
*Predicted Functions*	Predicted to be involved in stress-induced cell death regulation. LmjMCAs involved in cell cycle progression.	Regulation of cell death pathways; cytokinesis of parasite. Mechanistic role in PCD needs to be explored deeply.	*Pf*MCA-2 is likely involved in the progression of growth *in vitro* and indirectly responsible for cell death in the parasite (Vandana et al., 2018). Role of other MCAs of *P. vivax* and *P. berghei* needs to be elucidated.

### *Leishmania* Metacaspases

All *Leishmania* species express one single metacaspase gene except *L. infantum* and *L. donovani* species where two metacaspases have been reported (**Figures 2**–**4**). Structurally, they possess conserved Cys-His catalytic dyad; they also share an N-terminal domain containing putative mitochondrial localization signal along with less conserved proline-rich C-terminal domain probably involved in protein–protein interactions (Lee et al., 2007; Choi and Berges, 2013). The presence of functional N-terminal mitochondrial localization signal in *L. major* indicated that MCAs might indirectly affect mitochondrion to trigger cell death (Zalila et al., 2011). In *L. donovani*, *Ld*MCA-1 and *Ld*MCA-2 were found to be expressed in both promastigote and axenic amastigote forms of the parasite, whereas MCA of *L. mexicana* was located in the mitochondria and associated with the mitotic spindle (Castanys-Muñoz et al., 2012). Enzymatically, *Ld*MCAs displayed arginine/lysine substrate specificity without any auto-processing (Castanys-Muñoz et al., 2012). In contrast to *Ld*MCAs, *Lmj*MCA was activated by auto-processing but has the same arginine/lysine substrate specificity (González et al., 2007). Importantly, in *L. mexicana*, deletion of MCA did not affect the cell growth and viability of procyclic promastigotes (González et al., 2007). Further, overexpression of MCAs in amastigotes resulted in lower replication rate, which suggested that MCAs act as an amastigotes-specific growth suppressor.

Reports about the involvement of MCAs in *Leishmania* cell death reveal that *Lmj*MCA induces cell death in parasite, either by releasing its catalytic domain or by interaction of the C-terminal domain with partners involved in stress regulation or cell death in the presence of different apoptotic stimuli (miltefosine, curcumin, and H_2_O_2_) (Khademvatan et al., 2011; Casanova et al., 2015; Peña et al., 2017). Experimental evidence also suggested that the process of autophagy occurred in low nutrient concentration, which resulted in overexpression of *Lmj*MCA (Khademvatan et al., 2011). Further, *Lmj*MCA overexpression was found to enhance *L. major* sensitivity to oxidative stress compared to wild-type parasites expressing the endogenous metacaspases at optimum levels (Dolai et al., 2011). Report by Chowdhury et al. (2014) further suggested that the DiSB (topoisomerase type I-B inhibitor) induced apoptosis appears to be primarily associated with up-regulation in the expression level of metacaspases and generation of oxidative stress in *L. donovani* (Chowdhury et al., 2014). Interestingly, Shadab et al. (2017) group recently reported that peptide ecotin-like ISP3 of *L. major* specifically binds to MCAs and interferes with its trypsin-like activity in presence of heat shock, thereby significantly reducing parasite cell death (Shadab et al., 2017).

The endoplasmic reticulum-induced stress leads to the occurrence of Ca^+2^-dependent apoptosis-like cell death. However, cell death in ER stress-induced *Leishmania* cells was mediated by the mitochondrial apoptotic pathway. This pathwar involved ROS production, cytosolic Ca^+2^ imbalances, mitochondrial depolarization, and ultimately the release of Endo G from mitochondria to the nucleus via the cytoplasm (Dolai et al., 2011; Khademvatan et al., 2011). In addition, Khademvatan et al. (2011) report also suggested that the phenomenon of PARP cleavage (DNA repair enzyme that catalyzes the polyADP-ribosylation of various nuclear proteins), a prominent feature of metazoan apoptosis, also occurred in *Leishmania* (Khademvatan et al., 2011). Moreover, it was shown by a two-hybrid system that *L. major* mitogen-activated protein kinase MPK7 and calpain interact with the C-terminal domain of MCA, which probably causes induction of parasite death (Khademvatan et al., 2011; Shadab et al., 2017). Interestingly, miltefosine [hexadecylphosphocholine (HePc)] was found to be effective for visceral *leishmaniasis* and responsible for cleavage of PARP-like protein during apoptosis in *Leishmania* treated with H_2_O_2_. This process was blocked by caspase inhibitors (Khademvatan et al., 2011) (**Table 3** and **Figure 5**).

Studies revealed that LmjMCA is involved in autophagy in relation with over-expression of the gene and interaction of LmjMCA, mainly owing to its C-terminal domain, with itself and other proteins. These findings open new perspectives on the function of the MCA. The identification of the enzymatic substrates of LmjMCA and cell death triggering stimuli would clarify the metabolic pathways involving LmjMCA mediated cell death and/or autophagy. An important aspect of investigating metacaspases-like proteases in *Leishmania* is the uncertainty in autophagy/PCD relationships, which further leaves an open question about the mechanistic intersections between the two processes (Casanova et al., 2015; Chowdhury et al., 2014). Moreover, report by Castanys-Munoz et al. (2012) depicted that MCAs were not involved in *Leishmania* proliferation; rather, they act as amastigotes-specific growth suppressors, thereby regulating the parasite proliferation in the host. This self-regulation of growth is likely to be essential in maintenance of the infection thus greatly contributing to pathogenesis. Furthermore, stress-induced cell death was reported, which might be facilitated by the release of lysosomal enzymes after the disruption of organelle, and this could involve the cleavage of MCAs by the released cathepsin-like cysteine proteases. Conclusively, detailed studies on *Leishmania* metacaspases in context with the induction or inhibition route of apoptosis are an important subject to explore in depth.

### *Plasmodium* Metacaspases

Among the potential druggable targets against malaria, *Plasmodium* “metacaspases” are recently emerging as a potent candidate to explore. Till today, three MCAs were reported in the *Plasmodium* genomes: *P. falciparum* (*Pf*MCA1-3) and *P. vivax* (*Pv*MCA1-3) and in the murine parasite *P. berghei* (Atkinson et al., 2009) (*Pb*MCA1-3) (**Figures 2**–**4**). Proteins having caspase-like activity were identified in the cytoplasm of the ookinete, and more than 50% of the mosquito midgut stages of the parasite die naturally by apoptosis before the gut invasion (Meslin et al., 2007). Study by Picot et al. (1997) suggested that the *Pf*MCA-1 possesses the His-Cys catalytic dyad and upstream signaling pathways such as death domain or CARD, a module of 90–100 amino acids involved in apoptosis signaling pathways. The *Pf*MCA1-CARD consists of 76 amino acid residues that contributed the highest sequence similarity with caspase-1 (20.4%) of mice and humans (Meslin et al., 2007; Mutai and Waitumbi, 2010). Apoptosis-like DNA fragmentation/degradation in *P. falciparum* after chloroquine treatment *in vitro* was reported for the first time (Picot et al., 1997). Further, caspase-3 like subfamily member was associated with *P. berghei* ookinete apoptosis showing prominent features such as chromatin condensation, DNA fragmentation, and externalization of phosphatidylserine (Al-Olayan et al., 2002; Elmore, 2007). Moreover, in the presence of natural sunlight, there was an inhibition of parasite growth *in vitro*, which in turn leads to cell death in late trophozoites and schizonts (Engelbrecht and Coetzer, 2015). A study by Rathore et al. (2015) reported the occurrence of apoptosis-like cell death persuaded by cellular stress and organelle dysfunction, which was further attributed to the disruption of cellular homeostasis in *P. falciparum*. This report also demonstrated that the consistent stress on parasite led to the activation of Z-VAD-FMK-binding proteases and raised the cytosolic calcium levels along with the loss of mitochondrial membrane potential in parasites (Rathore et al., 2015). Activation of Z-VAD-FMK-binding proteases leads to degradation of the phylogenetically conserved protein, TSN (Tudor staphylococcal nuclease), a known target of metacaspases, along with the degradation of other components of the spliceosomal complex (Rathore et al., 2015). These findings are a clear indication for the existence of caspase-like proteases, which are known as “metacaspases” in *Plasmodium*, and they might have a role in PCD or in the regulation of growth and development of parasites (Ch’Ng et al., 2010; Rathore et al., 2015). However, the direct roles of these metacaspases are still not known in the malaria parasite (Shrestha and Megeney, 2012). Therefore, these proteases need to be explored in order to address their effective therapeutic potential against malaria.

**Figure 6:**
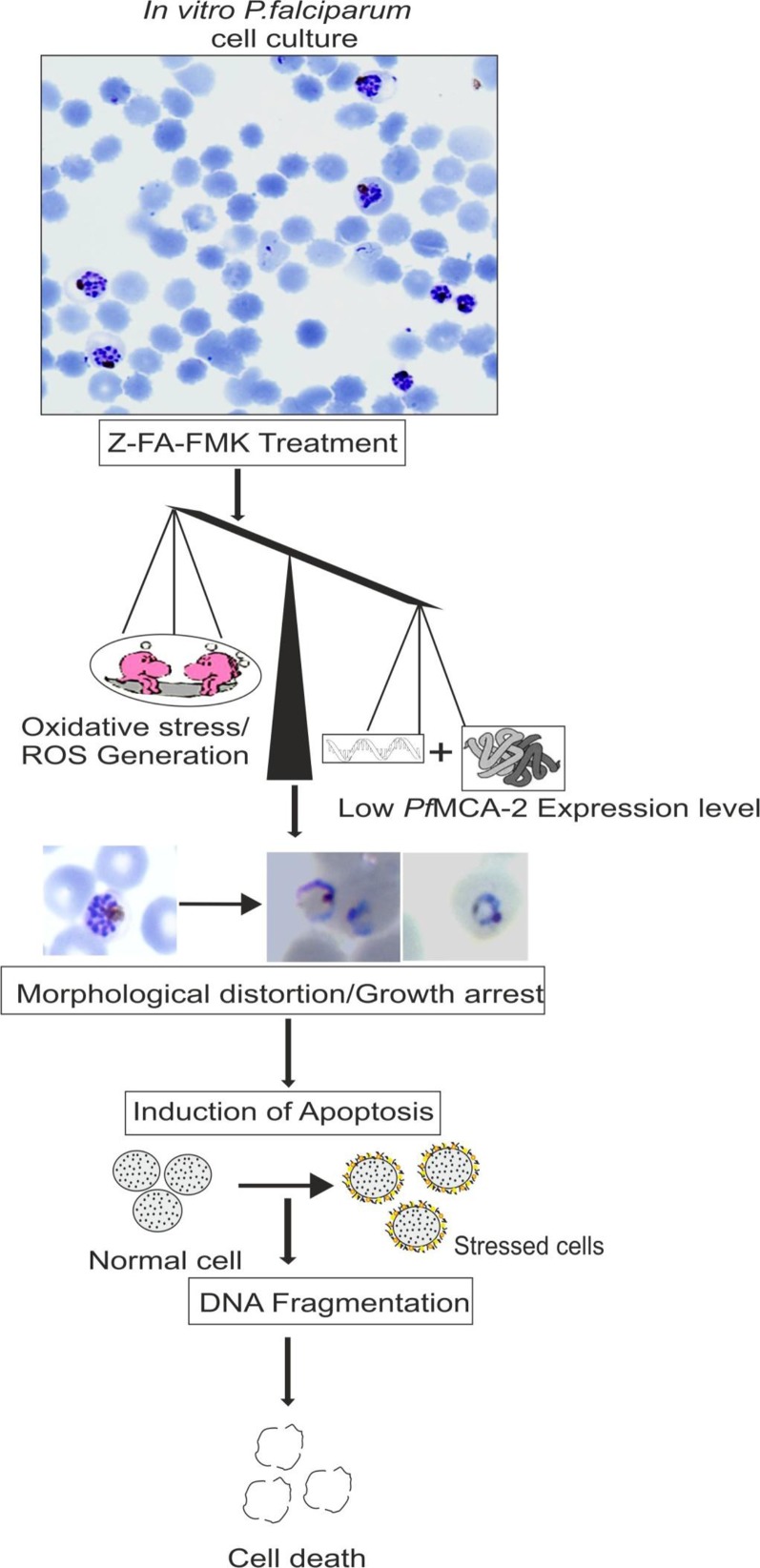
Schematic proposed model for *Pf*MCA-2 dependent apoptosis-like cell death *in viatro* induced by Z-FA-FMK (a known inhibitor of effector caspases) (Vandana et al., 2018).

In continuation of the above-mentioned reports on *Plasmodium* metacaspases, we find that *P. falciparum* MCA-2 (*Pf*MCA-2) have Arg/Lys substrate specificity at pH 7.4 (Vandana et al., 2018). A multiple sequence alignment of particular *Pf*MCA-2 has conserved tyrosine residues near to its cysteine and histidine catalytic dyad, which might be involved in substrate recognition. Unlike common malarial cysteine proteases such as falcipains, *Pf*MCA-2 cleaved neither hemoglobin nor BSA or casein-like macromolecular substrates. The effector caspases inhibitor Z-FA-FMK remarkably inhibits the *Pf*MCA-2 activity and the *in vitro* progression of *P. falciparum* (Vandana et al., 2018). Moreover, Z-FA-FMK also induces the oxidative stress by generating reactive oxygen species, which in turn is responsible for the occurrence of parasite cell death (Vandana et al., 2018) (**Figures 5** and **6**). This information could be important for further exploring the detailed function of metacaspases in *Plasmodium* (**Table 3** and **Figure 6**).

In summary, the studies done so far on this subject revealed that *Plasmodium* metacaspases might play a role in regulation of stress-dependent PCD and growth of the parasite.

However, *Plasmodium* metacaspases are not well studied in terms of their structural–functional prospects; therefore, more extensive research is needed in order to target such proteases for antimalarial drug discovery.

### Other Protozoan Parasite and Diatom Metacaspases

There are several studies that emphasize the occurrence of apoptosis-like cell death in other protozoan parasites. For instance, induction of apoptotic-like morphological changes in response to oxidative stress was observed in *Giardia, Trichomonas vaginalis, Entamoeba histolytica*, and *Blastocystis*. Bagchi et al. (2012) performed bioinformatics survey of *Giardia* genomes and reported key genes involved in autophagy (Bagchi et al., 2012). However, in the same study, the authors did not find any apoptotic gene that had significant similarity with identified apoptotic genes of other protozoa and eukaryotes except TOR and ATG8 (Bagchi et al., 2012). Moreover, the caspase-like activity in *Giardia* cell-free lysates was absent indicating the absence of caspase-like proteases in *Giardia* genome (Dubios et al., 2008; Bagchi et al., 2012). In *Toxoplasma gondii*, three metacaspases genes were annotated (TGGT1_206490, TGGT1_278975, and TGGT1_243298) and their respective proteins belong to ICE family (Li et al., 2016). Interestingly, only TGGT1_206490 carries histidine and cysteine catalytic dyad as reported for clan CD. However, such catalytic dyad was absent in the other two metacaspases. Study by Li et al. (2016) revealed that the *in vitro* growth and *in vivo* virulence of *T. gondii* were not affected in *Tg*MCA knockout parasite. However, there was a significant decline in apoptotic cell death in *Tg*MCA knockout parasites indicating *Tg*MCA plays an important role in *T. gondii* cell death (Li et al., 2016). Further, *Trichomonas vaginalis* also encodes metacaspases gene (TVAG_344040), but there are limited studies focusing its role in the cell death of the parasite (Chose et al., 2002). However, characteristic features of cell death including DNA fragmentation, chromatin condensation, nuclear fragmentation, vesicle formation, externalization of phosphatidylserine, vacuolization, etc. were reported in both *T. vaginalis* and *T. foetus* along with the typical caspase-like activity in *T. foetus* (Chose et al., 2002; Arroyo et al., 2015). Additionally, in *Blastocystis hominis*, caspase-3 like protease influenced the cell death, but it was not essential for the occurrence of DNA fragmentation/apoptosis (Nasirudeen and Tan, 2004). Moreover, the process of apoptosis in this unicellular parasite was associated with mitochondrial dysregulation. Similarly, the calpain-like protease was found to be responsible for inducing cell death in *Entamoeba histolytica* (Domínguez-Fernández et al., 2018). Further, metacaspases-5 from the model diatom *Phaeodactylum tricornutum* (PtMC5) possess calcium-dependent protease activity. This activity included auto-processing and cleavage after arginine residue (Van Creveld et al., 2018). Such type III metacaspase also controls cell death in a marine diatom. A study by Bidle and Bender (2008) further suggested that iron starvation leads to activation of metacaspases and PCD in the marine diatom *Thalassiosira pseudonana* (Bidle and Bender, 2008).

The main aim of focusing PCD associated proteins in protists is to target them for the development of a potential drug against the parasite-based infectious diseases. Therefore, at present, it is very important to explore the functional properties of such proteases for the elucidation of their functional relationship with metazoan caspases.

## Tudor Staphylococcal Nuclease as a Natural Substrate of Metacaspases

**Figure 7:**
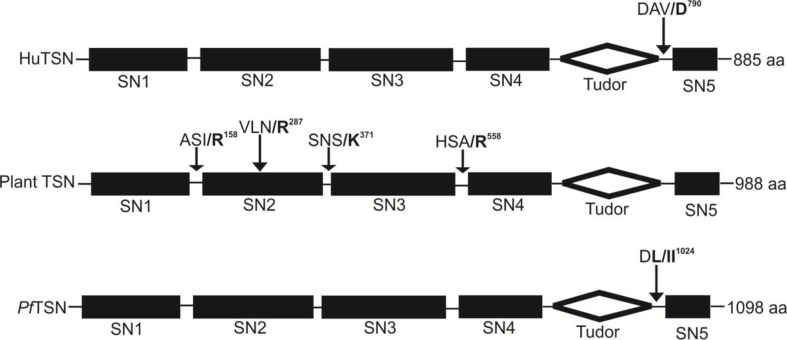
Domains organization of Tudor staphylococcal nuclease (TSN) in human (AAA80488), Plant (CAL38976) and *P. falciparum* (PF3D7_1136300) and location of MCAs cleavage sites were indicated by arrows. The cleavage of TSN occurred before the bold residues in all presented TSN proteins.

Unraveling the identity of natural substrates of metacaspases is important for revealing the molecular mechanisms of metacaspase-dependent processes. Presently, TSN is the only protein found to be cleaved by metacaspases *in vivo* (Gutierrez-Beltran et al., 2016). TSN is an evolutionarily and structurally conserved protein found in all eukaryotes (except for budding yeast). Functionally, TSN is involved in numerous fundamental mechanisms of gene regulation in animal cells, including transcription, mRNA splicing, and RNA silencing (Carmona-Gutierrez et al., 2010; Gutierrez-Beltran et al., 2010; Tsiatsiani et al., 2011). A report by Sundström et al. (2009) suggested that the reduced expression level of TSN causes cell death (Tsiatsiani et al., 2011; Bagchi et al., 2012) and impairs plant viability and stress tolerance (Carmona-Gutierrez et al., 2010). Moreover, active type II metacaspase from Norway spruce (mcII*-Pa*) causes fragmentation of endogenous TSN during developmental and oxidative stress-induced cell death. Further, caspase-3-mediated cleavage of human TSN at the cleavage site DAVD/S (Pop and Salvesen, 2009; Carmona-Gutierrez et al., 2010) has been revealed in cells undergoing apoptotic-like cell death. Further, Sundström et al. (2009) have demonstrated that TSN imparts stress tolerance in *Arabidopsis* through selective stabilization of mRNAs-encoding secreted proteins (Sundström et al., 2009). Notably, a significant proportion of these proteins are cysteine and serine protease inhibitors, which are known to suppress cell death in plants (Sundström et al., 2009; Dit Frey et al., 2010). But the mechanism of TSN-dependent stabilization of specific mRNAs remains unknown. Studies on the fragmentation of TSN by effector caspases and type-II metacaspase suggested that the animals and plants may have some common key proteolytic pathways responsible for the normal cell viability and physiology. A study by Rathore et al. (2015) suggested that *Pf*TSN harbors the DFVD motif near its C-terminus, which is a probable cleavage site for caspase-like enzymes (**Figure 7**). In the same study, authors have suggested that activation of caspase-like proteases leads to the reduction of the *Pf*TSN level in parasites along with other associated nuclear proteins like *Pf*SmD1 and *Pf*SmD3, which are part of nuclear splicing machinery (Rathore et al., 2015). We also found that *Pf*MCA-2 interacts specifically with the Tudor domain of the *Pf*TSN (Vandana et al., 2018). Further, the molecular mechanism of TSN degradation by *Plasmodium* MCA-2 and its consequence to the apoptosis pathway will be an interesting area to address in the near future. The existence of TSN and MCA interaction was not reported yet in *Leishmania* and *Trypanosoma.* Moreover, the correlation between metacaspase-mediated fragmentation of endogenous TSN and abolishment of its fragmentation by MCAs in the presence of inhibitor is an important area to investigate. In addition, the identification of protein targets other than P*f*TSN, which are cleaved by MCAs, and the molecular steps involved in activaltion of MCAs and TSN interaction will provide a fruitful avenue for future research.

## Therapeutic Potential of Metacaspases

The unusual properties of metacaspases such as their distributions, catalytic site substrate specificity, biological functions, and their absence in humans make them a potential drug target. Several studies have supported this concept with the demonstration that protease inhibitors have potent *in vitro* an *in vivo* antipathogenic effects. In recent years, understanding of the protease repertoire of parasite has remarkably increased due to the advancement in biochemistry and structural biology. Therefore, considering the specificity of metacaspases activity in protozoan parasites and their absence in humans opens a new path to describe potential activators or inhibitors specific to metacaspases. The miltefosine, currently used for treating leishmaniasis, acts as an apoptotic stimulus and induces apoptosis by targeting metacaspases. Further, a report by Kumar et al. (2018) suggested that the chemical molecule that can target the biosynthetic pathway of metacaspases is probably used as an effective anti-leishmanial agent (Kumar et al., 2018). Similarly, Basmaciyan et al. (2018)reported three apoptosis pathways involving *L. major* MCA (LmjMCA) and its potent inhibitors: i) an apoptosis pathway in which LmjMCA is activated (induced by miltefosine); ii) a pathway in which LmjMCA is inhibited (amphotericin B, curcumin, and H_2_O_2_); and iii) an LmjMCA-independent apoptosis pathway (pentamidine).

At present, it is not clearly known how protozoan metacaspases regulate the cell death in parasites, but it could be hypothesized that their conservation in protozoan presenting a long history of adaptation to environment reveals that metacaspases are key regulators of the parasite death or survival strategies. Therefore, in the near future, metacaspases could be considered as a potential drug target against parasite-based infectious diseases. Moreover, the development of potential inhibitors can either inactivate or activate the metacaspases pathway in order to favor parasite death either directly or through other mechanisms hindering one or several fundamental events caused by the metacaspases. In addition, inhibition of host cell apoptosis induced by a pathogen (Meslin et al., 2011) combined with induction of parasite apoptosis at the early stages of the infection is an integrative concept for future treatment that still needs to be elucidated deeply (**Figure 4**). Therefore, the approach for developing potent inhibitors against the parasite metacaspases, which can inhibit their activity further, can be useful for elucidating the therapeutic potential of protozoan metacaspases in detail.

## Conclusion

Parasites get benefits from several virulence factors such as proteases, enzymes, co-factors, etc. For example, *Plasmodium* proteases characterized so far are involved in hemoglobin degradation (Falcipains and Plasmepsins) (Silva et al., 1996; Shenai et al., 2000; Pandey et al., 2005), invasion (Subtilase, Plasmepsin IX & X) (Nasamu et al., 2017), egress and breakdown of RBC (SUB1 & Plasmepsin II, X), trafficking pathway (Plasmepsin V) (Russo et al., 2010), and many more, which help parasites to survive in an intra- or extracellular host environment. Similarly, other parasite proteins such as *Leishmania* substilisin-like serine protease, which plays a role in promastigotes to amastigotes differentiation (Da Silva-Lopez et al., 2005), Presenilin 1 protease, involved in autophagy in L. major, which is involved in autophagy in *L. major* (Besteiro et al., 2007), Cruzain (cysteine protease) of T. cruzi, which helps in immune evasion (Doyle et al., 2011), etc., are found to be important for parasite physiology. Therefore, proteases are considered to be potent drug targets against parasites-based infectious diseases (Nuñez et al., 1998; Deponte, 2008). In this context, the function of non-metazoan metacaspase-like proteases needs to be deeply explored as some recent findings suggest that these proteases are also important for the maintenance of physiology along with death and survival strategies of parasites (Jiménez-Ruiz et al., 2010; Deu, 2017). For instance, *Trypanosoma* and *Leishmania* metacaspases are increasingly being implicated as important players of PCD. Similarly, our recent report on *Pf*MCA-2 reveals that the optimum level of *Pf*MCA-2 was essential for parasite growth and cell viability. Further, our report also suggests that *Pf*MCA-2 is important for stress regulation inside the cell (Vandana et al., 2018). Elucidating the mechanism behind the metacaspase-associated PCD is an important area of research, and it provides opportunities to target these proteases for potential drug discovery against parasite infectious diseases. It was also observed that specific metacaspase inhibitors disorient parasite morphology and reduce the cell viability and growth arrest, and also cause circumstantial cell death. Hence, such specific inhibitors will play an important role to understand the physiological significance of metacaspases-like proteases in disease pathogenesis and to identify them as promising candidates for drug development.

## Data Availability

All datasets generated for this study are included in the manuscript and the supplementary files.

## Author Contributions

KV and KP developed the idea, collected information, interpreted and reviewed the literature and wrote the article. RD, AK, and RT helped in the writing process. All the authors approved and reviewed the final version of the review article.

## Funding

This work was supported by the Intramural grant of ICMR-NIMR and by the Ramalingaswami Fellowship, BT/HRD/35/02/2009, DBT sanctioned to KCP.

## Conflict of Interest Statement

The authors declare that the research was conducted in the absence of any commercial or financial relationships that could be construed as a potential conflict of interest.
